# Extrapolation errors in Liu et al.’s CAM integrative review of health care professionals in New Zealand

**DOI:** 10.1186/s12906-023-04259-3

**Published:** 2024-05-13

**Authors:** Jillian Marie McDowell, Susan Heather Kohut, Debra Betts

**Affiliations:** 1 Prohealth Physiotherapy, 124 Kelvin St, Invercargill, New Zealand; 2 School of Clinical Sciences, Faculty of Health and Environmental Sciences, Auckland University of Technology, Auckland, New Zealand; 3 New Zealand School of Acupuncture and Traditional Chinese Medicine, Wellington, New Zealand

**Keywords:** Physiotherapy, Acupuncture, Safety, Extrapolation, Survey, Error

## Abstract

This letter is to highlight errors made by Liu et al. in their 2020 paper in BMC Complementary Medicine and Therapies, “Complementary and alternative medicine—practice, attitudes, and knowledge among healthcare professionals in New Zealand: an integrative review”. Substantial errors in their citation of the recent research and methodology by McDowell, Kohut & Betts (2019) pertaining to the practice of acupuncture in New Zealand by physiotherapists are presented. The actual results of McDowell et al.’s work and the true state of acupuncture use by their sample group is reported.

## Background

This letter is to highlight errors made by Liu et al. in their 2020 paper in BMC Complementary Medicine and Therapies, “Complementary and alternative medicine—practice, attitudes, and knowledge among healthcare professionals in New Zealand: an integrative review” [1], when citing the paper “Safe acupuncture and dry needling during pregnancy: A survey of New Zealand physiotherapists’ practice”, published in 2019 in Integrative Medicine Research [2]. Extrapolation errors are evident in their abstract, results and discussion’s statistics. Furthermore, the methodology has been mis-cited, as has interpretation of postgraduate physiotherapy acupuncture training in New Zealand (NZ).

### Extrapolation errors – statistical

In Liu et al.’s abstract on complementary and alternative medicine (CAM) they state:

“When treating pregnant women, *48.4% of physiotherapists practise acupuncture*, and 37.3% of midwives recommend CAM” page 1 [1].

These statistics are repeated in their results section for prevalence of practice, use and referral for CAM:

“Findings indicated that around 25% of GPs practise CAM, and 82.3% refer patients to CAM practitioners. *When treating pregnant women, 48.4% of physiotherapists practise acupuncture,* and 37.3% of midwives recommend CAM” page 4 [1],

and again, in their discussion:

“Findings indicated that CAM use is common among New Zealand healthcare professionals: around 25% of GPs practise CAM, and 82.3% refer patients to CAM practitioners; *when treating pregnant women, 48.4% of physiotherapists practise acupuncture,* and 37.3% of midwives recommend CAM” page 7 [1].

More accurately McDowell et al. [2] reported that 60 physiotherapists (48.4% of the 124 total respondents) practise acupuncture during pregnancy. This represented 0.8% of the 7337 registered physiotherapists in NZ. Liu et al.’s citation would infer that 48.4% of all registered physiotherapists in NZ (3551/7337) practice acupuncture for pregnant women; a 60-fold overestimation. McDowell et al. very clearly reported the limitations of their study;

“The results of this purposive study may not be indicative of the opinion of all NZ physiotherapy acupuncturists and dry needlers” page 37 [2] ,

and Liu et al.’s interpretative flaws may lead to future publication errors if it is cited further.

### Extrapolation error—Post graduate training

The following statement in Liu et al.’s paper is inaccurate and misleading.

“Findings indicated that approximately one quarter of GPs have received formal training in CAM, *while 44.4% of physiotherapist received (postgraduate) training in acupuncture”* page 6 [1].

More correctly 53% of physiotherapists (*n* = 32) of those who answered in the affirmative to McDowell et al.’s survey, (i.e., who would provide acupuncture during pregnancy), had undergone post-graduate training with a resulting university-based qualification. It is important to note that physiotherapists choose to study and practice acupuncture in NZ as a postgraduate competency. Yet again Liu et al. have committed a statistical error of extrapolating a very small survey finding to the entire NZ physiotherapy population.

### Methodology citation error

In their paper Liu et al. also state:

“Instruments used in the eight surveys varied, without psychometric properties reported; *four studies used instruments that were developed and adapted from previous studies”*. page 4 [1].

McDowell et al.’s paper clearly describes the development of the electronic survey questionnaire, expert peer review, and piloting prior to the main survey. It was not adapted from a previous study as cited by Liu et al. above.

## Discussion

### The current state of acupuncture practice in NZ by physiotherapists

The practice of acupuncture is not currently regulated for physiotherapists in NZ, being considered “a modality within the practice of physiotherapy” [3]. Registered physiotherapists may practice within a defined field of practice as autonomous practitioners but must “complete or be currently undertaking, relevant, sufficient, and appropriate education and training and continuing professional development to maintain such knowledge and competence” [4]. NZ registered physiotherapists who have gained post graduate qualifications in acupuncture or have trained in dry needing are termed “physiotherapy acupuncturists” to identify as a separate profession to acupuncturists.

At the time of the survey 7337 physiotherapists were registered to practice in NZ [5]. Sixty nine percent of those were members of their national society, Physiotherapy New Zealand (PNZ) [6], and only 310 were members of PNZ’s special interest group the Physiotherapy Acupuncture Association of New Zealand (PAANZ). Further demographic information on the total number of NZ physiotherapists who have undertaken relevant education or who are currently practicing needling was not available.

Acupuncture has been taught and practiced by physiotherapists in NZ since 1992 [7–9]. However, the total number of physiotherapists utilising this complementary and alternative medicine in NZ is unknown. This has been a limiting factor for several research surveys to date [10, 11]. The paper “Safe acupuncture and dry needling during pregnancy: New Zealand physiotherapists’ opinion and practice”, was published in 2019 in Integrative Medicine Research [2]. It is important to emphasise that the paper’s results did not have access to the entire population of potential respondents. The Privacy Act of New Zealand [12] prohibits the direct use of Physiotherapy Board of New Zealand (PBNZ) membership lists to target the entire survey population. McDowell et al.’s survey recruitment was via invitation only by PNZ e-mail, with additional links available through PAANZ and PNZ webpages, closed acupuncture group Facebook pages and physiotherapy newsletters within NZ.

Whilst McDowell et al.’s primary aim was to gather basic demographics and examine the opinions, practice and level of understanding held by NZ physiotherapy acupuncturists providing acupuncture and dry needling for pregnant women, their results were never intended to be reflective of the entire population of physiotherapists in New Zealand.

Regrettably, this fact seems to have been lost by Liu et al. when extrapolating McDowell et al.’s data as verbatim statistics for the entire NZ physiotherapy population.

Survey response rates have been on the decline for the past decade in the field of health related research [13, 14], with response rates consistently lower than those of surveys of the general population [15]. Field et al. [16] reported response rates of between 13 and 39% are common in healthcare research. Cunningham et al. [13] found an overall response rate of 35% amongst physicians in Canada on a sensitive topic (medical billing practices) which was comparable to 39.6% response rate reported in the meta-analysis by Cook [17]. The McDowell et al.’s survey noted the existence of a low response rate (particularly from physiotherapy acupuncturists with only dry needling training), and a high number of missing responses within individual responses. This sample may not represent any definable population larger than NZ physiotherapy acupuncturists, a concept that appears to have been neglected by Liu et al.’s integrative review [1].

### Further research

Research-based practice is central to improving the quality of health care by obtaining information about the knowledge, attitudes, practice patterns, the needs of practitioners [18] and to evaluate the impact of clinical research on practice [13]. Soliciting practitioner input is also essential when existing healthcare policies are being updated or to inform new policies [19].

Liu et al. have failed to recognize the limitations of McDowell et al.’s research sample, thus committing a statistical error of extrapolating a small survey finding to the entire NZ physiotherapy population. This raises concern that others may read their paper and cite it further (especially with the error prominent in the abstract), compounding errors in perceived practice if they are compared to the inflated NZ data. To date four papers have cited Liu et al.’s paper [20–23], fortunately without specifically referring to the erroneous sections. We ask that Lui et al.’s statements be corrected via publication of this correspondence, that is indexed and bidirectionally linked to the original article to prevent further and future errors.

## Data Availability

Not applicable.
